# Supplementary material for: Tract-specific analysis improves sensitivity of spinal cord diffusion MRI to cross-sectional and longitudinal changes in amyotrophic lateral sclerosis
Source: Commun Biol. 2020 Jul 10;3:370. doi: 10.1038/s42003-020-1093-z (PMC7351722; doi:10.1038/s42003-020-1093-z)
Supplement: Supplementary file 1 — Supplementary information [file 42003_2020_1093_MOESM1_ESM.pdf]

Supplementary information

Supplementary Figures

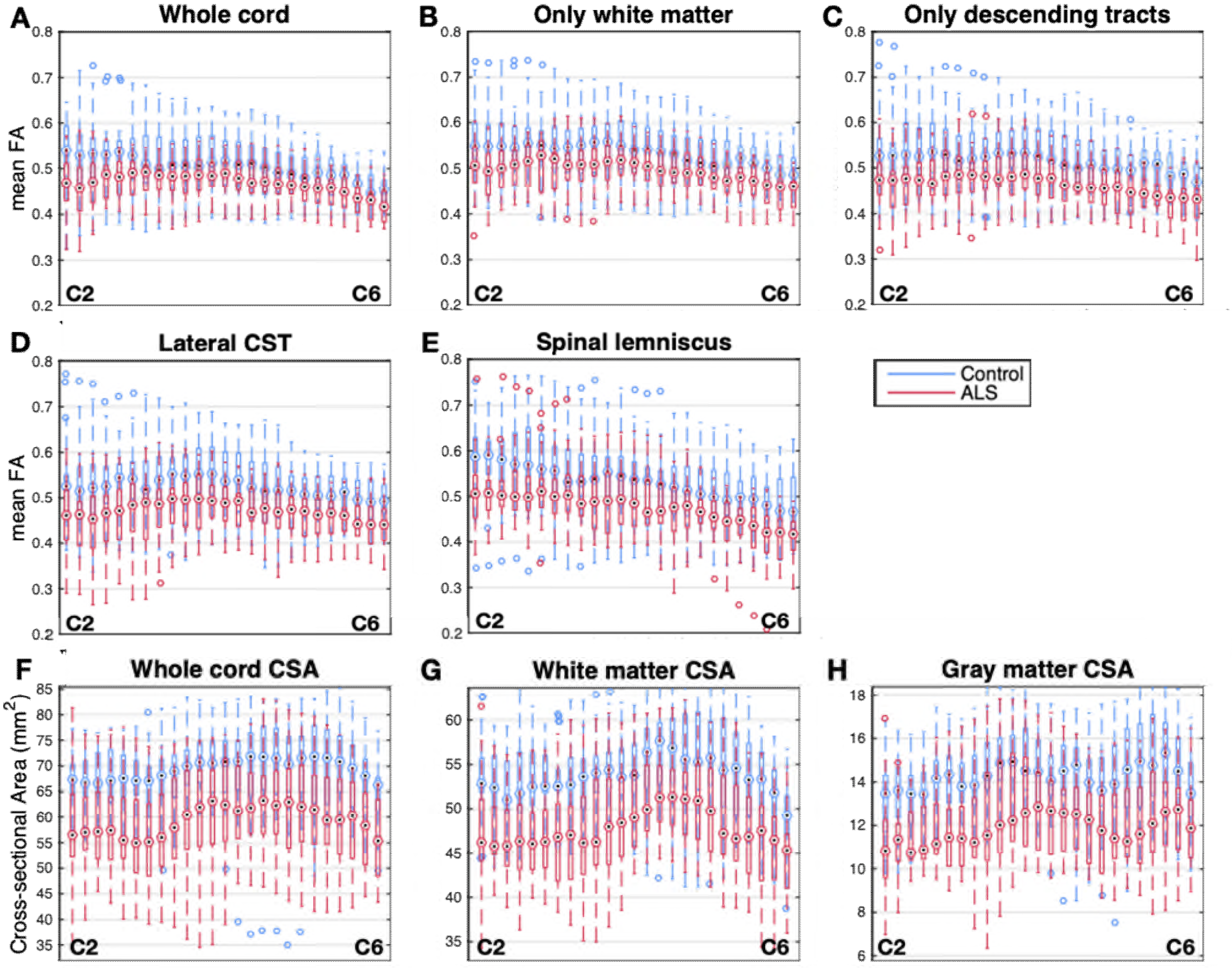

Supplementary figure 1. Box plots (corresponding to the data shown in Fig. 2-4 in the main paper), showing cross-sectional differences in FA along (A) whole cord, (B) only white matter tracts, (C) only descending tracts, (D) lateral CST, and (E) spinal lemniscus, and CSA along (F) whole cord, (G) only white matter, and (H) only gray matter, between ALS (n=20) and control (n=20) participants. The x-axis shows the variation from C2 to C6. The box plot at each point shows the median (circle with dot), 25th and 75th percentiles (edges of the box), and the range of FA / CSA (the whisker end points) excluding the outliers (individual circles).

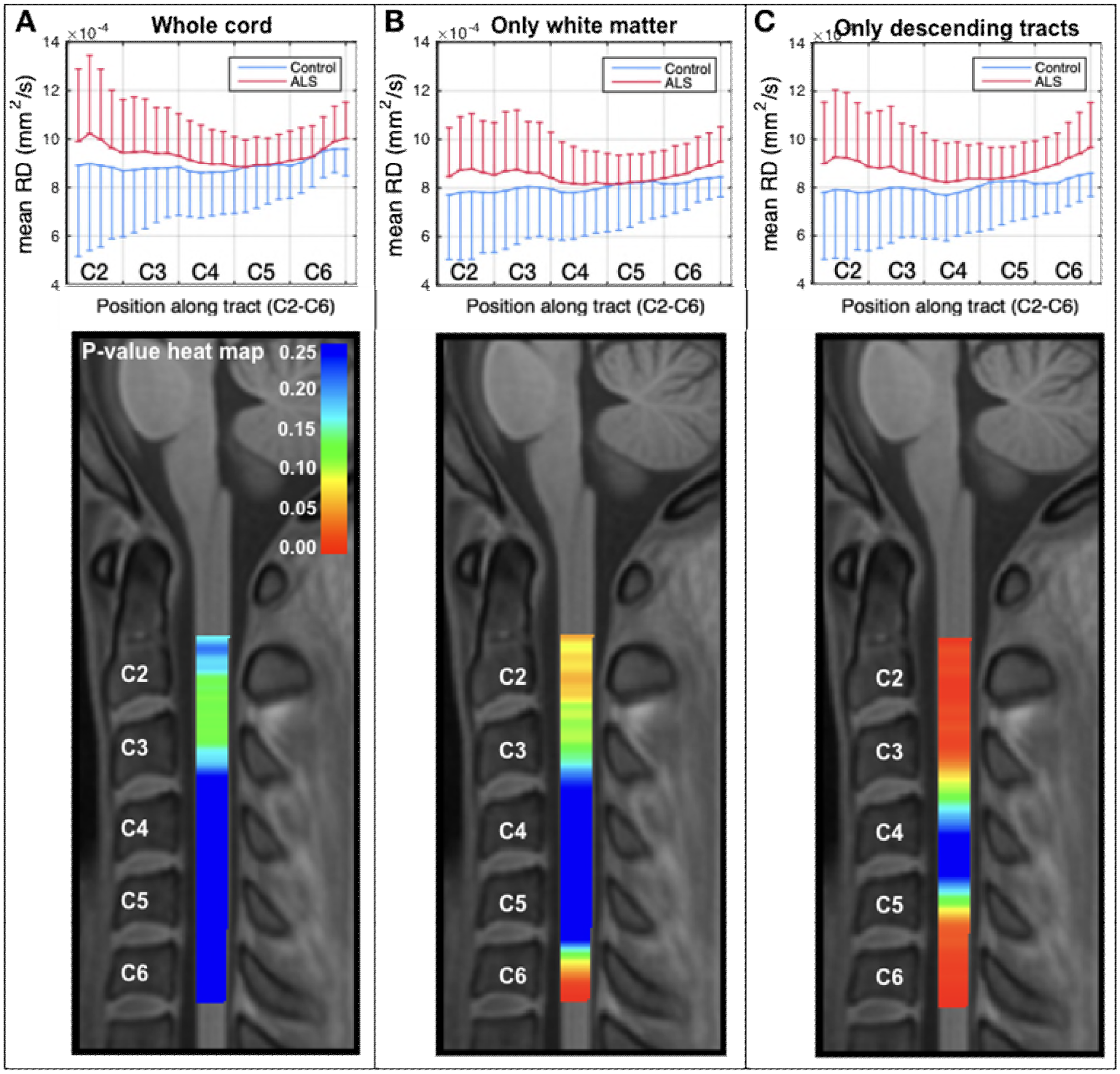

Supplementary figure 2. Graphical representations of cross-sectional differences in RD between ALS (n=20) and control (n=20) participants (the one-sided error bars represent standard deviation), and corresponding *p*-value heat maps, highlighting spinal level with significant group differences (colors orange to red show areas where significant difference exists). Three cases, whole cord without segmentation, only white matter, and only descending tracts, are shown. The sensitivity of the method increases with the segmentation.

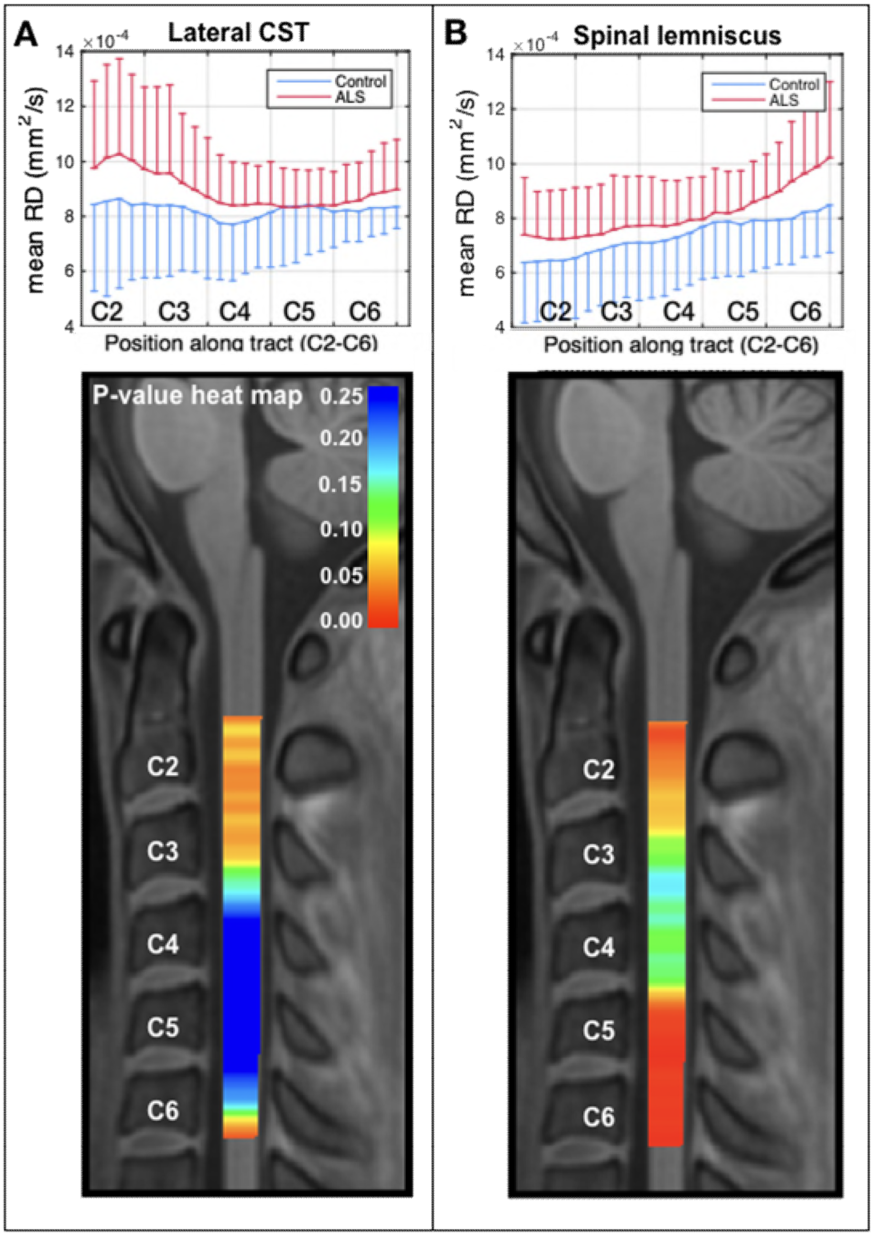

Supplementary figure 3. Graphical representations of cross-sectional differences in RD between ALS (n=20) and control (n=20) participants (the one-sided error bars represent standard deviation), and corresponding *p*-value heat maps, highlighting spinal level with significant group differences for lateral CST and spinal lemniscus (colors orange to red show areas where significant difference exists).

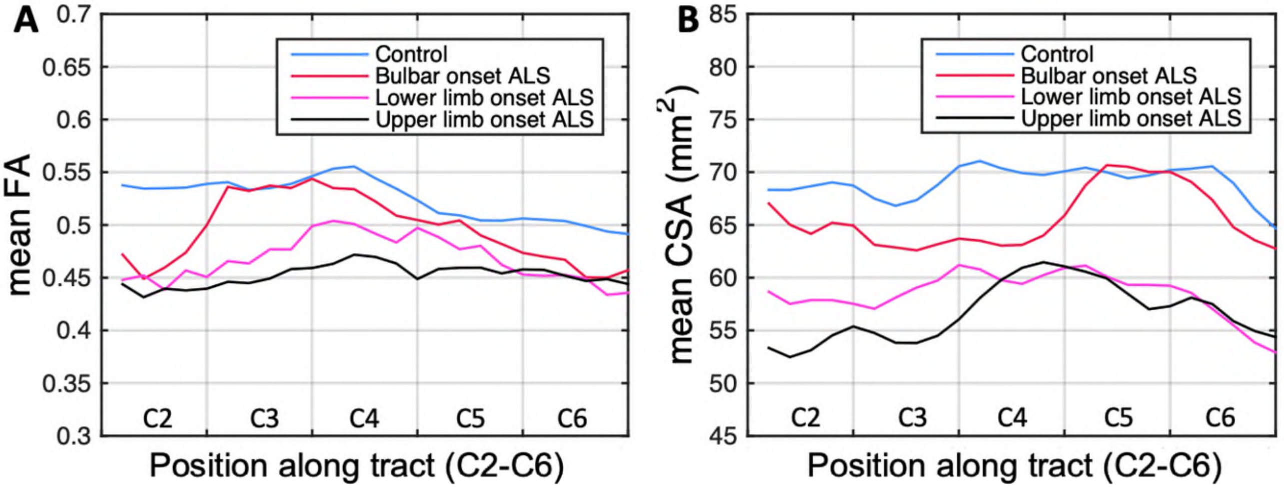

Supplementary figure 4. Comparison between baseline cross-sectional group difference in (A) FA along the lateral CST, and (B) CSA of the whole cord of 20 control, 5 bulbar onset ALS, 5 lower limb onset ALS, and 10 upper limb onset ALS participants.

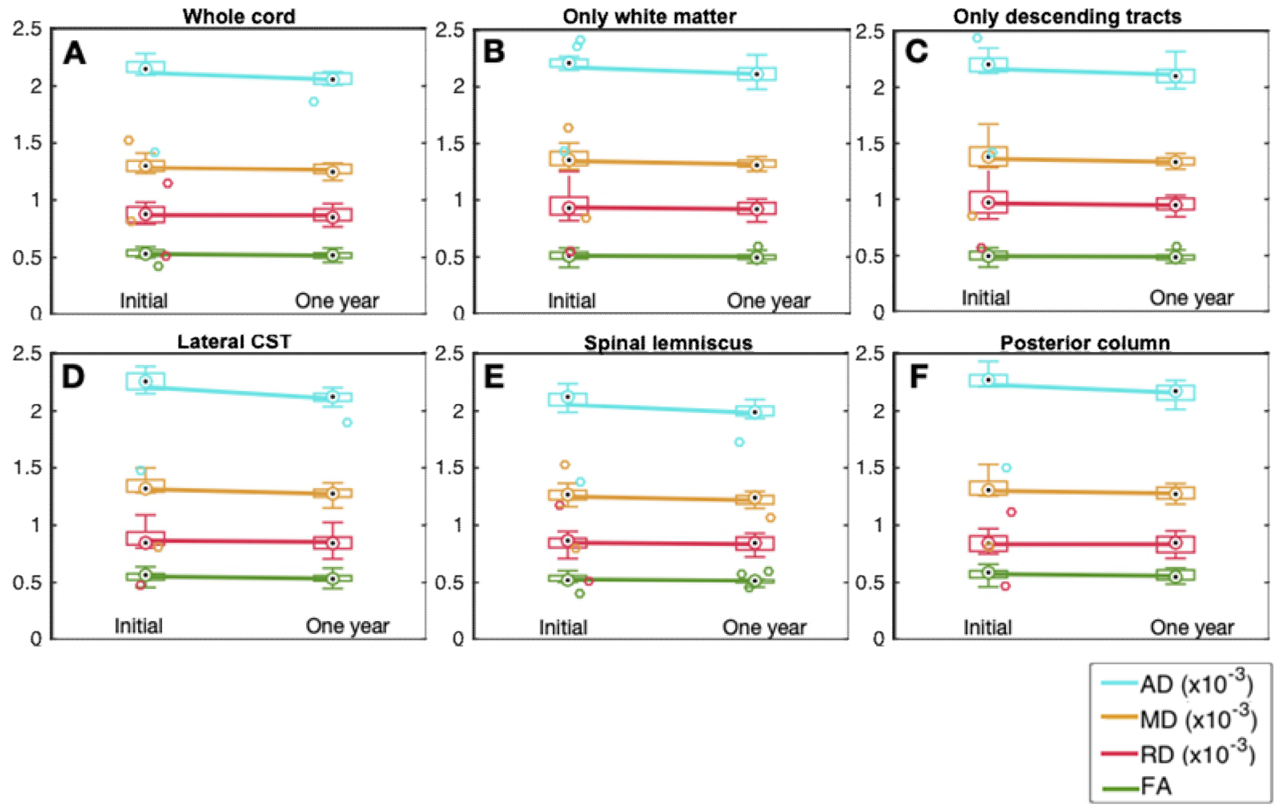

Supplementary figure 5. Longitudinal changes in control participants (n=13), in FA, RD, MD, and AD (averaged across C2-C6) in (**A**) whole spinal cord, (**B**) spinal white matter, (**C**) descending tracts, (**D**) lateral CST, (**E**) spinal lemniscus, and (**F**) posterior columns. No significant change is noted in any of the metrics. The line between initial and one year data connects the means. The box plots show the median (circle with dot), 25th and 75th percentiles (edges of the box), and the range of FA / CSA (the whisker end points) excluding the outliers (individual circles).

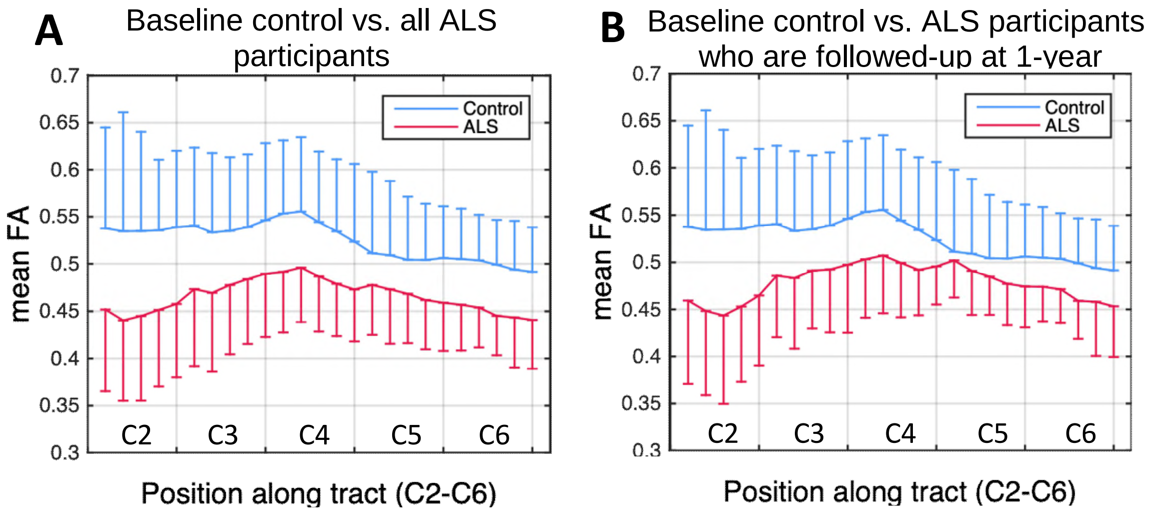

Supplementary figure 6. Comparison between baseline cross-sectional group difference in FA along the lateral CST of (A) 20 control and 20 ALS subjects, and that of (B) 20 controls and 11 ALS subjects followed-up at one-year. Both demonstrate a significant reduction in mean FA from C2 to C3.
