# Supplementary material for: Tract-specific analysis improves sensitivity of spinal cord diffusion MRI to cross-sectional and longitudinal changes in amyotrophic lateral sclerosis
Source: Commun Biol. 2020 Jul 10;3:370. doi: 10.1038/s42003-020-1093-z (PMC7351722; doi:10.1038/s42003-020-1093-z)
Supplement: Supplementary file 2 — Reporting Summary [file 42003_2020_1093_MOESM2_ESM.pdf]

nature research

Corresponding author(s): Pramod K Pisharady
Last updated by author(s): May 20, 2020

# Reporting Summary

Nature Research wishes to improve the reproducibility of the work that we publish. This form provides structure for consistency and transparency in reporting. For further information on Nature Research policies, see Authors & Referees and the Editorial Policy Checklist.

## Statistics

For all statistical analyses, confirm that the following items are present in the figure legend, table legend, main text, or Methods section.

| n/a | Confirmed | |
|---|---|---|
| ☐ | ☒ | The exact sample size (*n*) for each experimental group/condition, given as a discrete number and unit of measurement |
| ☐ | ☒ | A statement on whether measurements were taken from distinct samples or whether the same sample was measured repeatedly |
| ☐ | ☒ | The statistical test(s) used AND whether they are one- or two-sided<br>*Only common tests should be described solely by name; describe more complex techniques in the Methods section.* |
| ☐ | ☒ | A description of all covariates tested |
| ☐ | ☒ | A description of any assumptions or corrections, such as tests of normality and adjustment for multiple comparisons |
| ☐ | ☒ | A full description of the statistical parameters including central tendency (e.g. means) or other basic estimates (e.g. regression coefficient) AND variation (e.g. standard deviation) or associated estimates of uncertainty (e.g. confidence intervals) |
| ☐ | ☒ | For null hypothesis testing, the test statistic (e.g. $F$, $t$, $r$) with confidence intervals, effect sizes, degrees of freedom and $P$ value noted<br>*Give P values as exact values whenever suitable.* |
| ☒ | ☐ | For Bayesian analysis, information on the choice of priors and Markov chain Monte Carlo settings |
| ☒ | ☐ | For hierarchical and complex designs, identification of the appropriate level for tests and full reporting of outcomes |
| ☒ | ☐ | Estimates of effect sizes (e.g. Cohen's *d*, Pearson's *r*), indicating how they were calculated |

*Our web collection on statistics for biologists contains articles on many of the points above.*

## Software and code

Policy information about availability of computer code

| | |
|---|---|
| Data collection | Data acquisition softwares supplied with Siemens Trio and Prisma scanners |
| Data analysis | Our code is developed in Matlab software, we used MATLAB 8.4 for running the code.<br>We used FSL version 5.0.7 for preprocessing of the data and for the diffusion tensor model fitting.<br>We used the spinal cord toolbox (SCT) version 3.1.0 for the spinal cord segmentation.<br>We used the open-source FADTTS toolbox version 5.01 for the varying coefficient based analysis of the data. |

For manuscripts utilizing custom algorithms or software that are central to the research but not yet described in published literature, software must be made available to editors/reviewers. We strongly encourage code deposition in a community repository (e.g. GitHub). See the Nature Research guidelines for submitting code & software for further information.

## Data

Policy information about availability of data

All manuscripts must include a data availability statement. This statement should provide the following information, where applicable:

- Accession codes, unique identifiers, or web links for publicly available datasets
- A list of figures that have associated raw data
- A description of any restrictions on data availability

Data availability:
Deidentified data will be made available on request for the purposes of reproducing the results presented, subject to institutional approval.
Code availability:
The code used to produce the results reported in this article is available from the corresponding author upon reasonable request.

# Field-specific reporting

Please select the one below that is the best fit for your research. If you are not sure, read the appropriate sections before making your selection.

☒ Life sciences ☐ Behavioural & social sciences ☐ Ecological, evolutionary & environmental sciences

For a reference copy of the document with all sections, see nature.com/documents/nr-reporting-summary-flat.pdf

# Life sciences study design

All studies must disclose on these points even when the disclosure is negative.

| | |
|---|---|
| Sample size | We tested for location-specific differences along the spinal cord, and not just whole-cord averaged metrics. There is no straightforward sample size calculation formula in this case, for the varying coefficient model that we have used. Also, as there is no prior 'tract-specific' along the cord data available, a simulation study would need to be designed and carried out to obtain an approximate sample size. Such simulation studies typically perform best when there is some pilot data available, on which to base them. We could perform a standard sample size calculation post-hoc for progression of e.g. within-patient whole-cord averaged DTI metrics, but this will not show the statistical power of the method we used (varying coefficient model). |
| Data exclusions | We originally planed to analyze the data from vertebral levels C2-C7 of the spinal cord. However the data from C7 is excluded for ALL the subjects, due to the partial coverage of C7 in some of the subjects. This is explained in the Methods section. |
| Replication | The results and findings we report can be reproduced as they are obtained using computer code operating on MRI data. However, we have not included "test-retest" reproducibility data [NOTE: we have this data from our FRDA study if needed]. |
| Randomization | This is a group analysis comparing patients with healthy controls. People who met revised El Escorial Criteria for clinically possible, probable, or definite ALS were recruited to the patient population. Healthy volunteers were recruited as controls from the general public and selected to match ALS participants’ age range and sex frequency. |
| Blinding | The disease status of the subjects were known to the investigators as the objective was to find the differences between the two groups (patients and controls), given their status. |

# Reporting for specific materials, systems and methods

We require information from authors about some types of materials, experimental systems and methods used in many studies. Here, indicate whether each material, system or method listed is relevant to your study. If you are not sure if a list item applies to your research, read the appropriate section before selecting a response.

## Materials & experimental systems

| n/a | Involved in the study | |
|---|---|---|
| ☒ | ☐ | Antibodies |
| ☒ | ☐ | Eukaryotic cell lines |
| ☒ | ☐ | Palaeontology |
| ☒ | ☐ | Animals and other organisms |
| ☐ | ☒ | Human research participants |
| ☒ | ☐ | Clinical data |

## Methods

| n/a | Involved in the study | |
|---|---|---|
| ☒ | ☐ | ChIP-seq |
| ☒ | ☐ | Flow cytometry |
| ☐ | ☒ | MRI-based neuroimaging |

## Human research participants

Policy information about studies involving human research participants

| | |
|---|---|
| Population characteristics | The ALS cohort was on average an early-stage cohort with mean ALSFRS-R of 40.0, with 60% in King's Stage 1 or 2. This proportion was reduced to 45.5% at the one-year follow-up visit. As per revised El Escorial Criteria, there were 7 possible, 8 probable, and 5 definite subjects at baseline. Out of the 7 possible subjects, 1 progressed to definite, 2 progressed to probable, 3 remained as possible, and 1 was withdrawn from the study at the one-year follow-up. Out of the 8 probable subjects, 3 remained as probable and 5 were withdrawn from the study. Only two out of the five definite subjects returned for the one-year follow-up and they remained in the definite status. The mean change in ALSFRS-R of ALS participants at the one-year follow-up visit was -4.8 points, with an average slope of -0.4 points/month. There were four deaths in the ALS cohort and no deaths in the control cohort during the study. |
| Recruitment | People who met revised El Escorial Criteria for clinically possible, probable, or definite ALS were recruited from the ALS Association Certified Treatment Centers of Excellence at the University of Minnesota and Hennepin County Medical Center. Healthy control volunteers were recruited from the general public and selected to match ALS participants’ age range and sex frequency. Exclusion criteria included the presence of neurologic illnesses other than ALS, the inability to tolerate MRI scanning, and the failure to meet MRI safety criteria. |

Ethics oversight | Institutional Review Board, University of Minnesota

Note that full information on the approval of the study protocol must also be provided in the manuscript.

# Magnetic resonance imaging

## Experimental design

Design type: Microstructural analysis using diffusion MRI

Design specifications: Subjects were scanned at baseline, 6 months, and 12 months.

Behavioral performance measures: There were no performance measures during the MRI data acquisition. The functional impairment in ALS participants was measured using the ALS Functional Rating Scale-Revised (ALSFRS-R), before the MRI data acquisition. Cognitive and behavioral status was also assessed using the Edinburgh Cognitive Behavioral ALS Screen (ECAS).

## Acquisition

Imaging type(s): diffusion MRI

Field strength: 3T

Sequence & imaging parameters: The data were obtained using the RESOLVE sequence, with diffusion gradients along 30 directions and a b-value of 650 s/mm2. Six additional volumes without diffusion encoding were equally interleaved in the dataset yielding a total of 36 volumes. We obtained 30 slices with thickness 3.3 mm and voxel size 1.12x1.12 mm2 (FoV=118x62). Two sets of data were collected during each session, with reversed phase encoding directions (anterior to posterior and posterior to anterior).

Area of acquisition: C-spine

Diffusion MRI: [x] Used [ ] Not used

Parameters: 30 diffusion directions, b-value 650 s/mm2, six b0 volumes, 30 slices with thickness 3.3 mm and voxel size 1.12x1.12 mm2.

## Preprocessing

Preprocessing software: We used FSL version 5.0.7 for preprocessing of the data and for the diffusion tensor model fitting.
We used the spinal cord toolbox (SCT) version 3.1.0 for the spinal cord segmentation.

Normalization: Particular normalization of the data was not required (except for the below structural registration) as the features are extracted after diffusion tensor model (DTI) fitting.

Normalization template: The spinal cord toolbox uses a multimodal template of the spinal cord, created from 50 healthy subjects (PAM50), aligned with the ICBM152 space, to register the data before segmentation.

Noise and artifact removal: The data were corrected for distortions due to eddy currents, susceptibility-induced off-resonance artifacts and subject motion using FSL version 5.0.7.

Volume censoring: No volume censoring was done

## Statistical modeling & inference

Model type and settings: We used a varying coefficient model for the analysis. We conducted both univariate and multivariate analysis (further details are provided in the statistical analysis section of the paper).

Effect(s) tested: The effect of disease on cord cross-sectional area and microstructural changes measured with DTI features

Specify type of analysis: [ ] Whole brain [ ] ROI-based [x] Both

Anatomical location(s): We analyzed both the whole cord as well as all possible segmentations of the cord.

Statistic type for inference (See Eklund et al. 2016): Our analysis is 'tract-wise', not cluster-wise.

Correction: The p-values were corrected for multiple testing along the cord, with a wild bootstrap method (see reference 12 in the paper for details).

## Models & analysis

| n/a | Involved in the study |
| --- | --- |
| ☒ | ☐ Functional and/or effective connectivity |
| ☒ | ☐ Graph analysis |
| ☐ | ☒ Multivariate modeling or predictive analysis |

| | |
| --- | --- |
| Multivariate modeling and predictive analysis | The independent variables are the diffusion metric fractional anisotropy (extracted through DTI model fitting) and a morphometric measure, the cord cross-sectional area. |
