# Supplementary material for: Tract-specific analysis improves sensitivity of spinal cord diffusion MRI to cross-sectional and longitudinal changes in amyotrophic lateral sclerosis
Source: Commun Biol. 2020 Jul 10;3:370. doi: 10.1038/s42003-020-1093-z (PMC7351722; doi:10.1038/s42003-020-1093-z)
Supplement: Supplementary file 4 — Description of Additional Supplementary Files [file 42003_2020_1093_MOESM4_ESM.pdf]

Description of additional Supplementary Items

Supplementary Data 1. Source data used to generate the charts present in the manuscript.
